# Comprehensive Review of the Psychosocial Impact on Parents of Newborns With Congenital Heart Disease: A Significant Problem in Low- and Middle-Income Countries

**DOI:** 10.7759/cureus.68532

**Published:** 2024-09-03

**Authors:** Sorin Vasilescu, Diana Iulia Vasilescu, Adriana Mihaela Dan, Octavian Munteanu, Mihaly Enyedi, Iulian M Slavu, Monica Mihaela Cirstoiu

**Affiliations:** 1 Department of Obstetrics, Gynecology, and Neonatology, University Emergency Hospital Bucharest, Bucharest, ROU; 2 Department of Medicine, Carol Davila University of Medicine and Pharmacy, Bucharest, ROU; 3 Department of Anatomy, Carol Davila University of Medicine and Pharmacy, Bucharest, ROU; 4 Department of Radiology, Carol Davila University of Medicine and Pharmacy, Bucharest, ROU; 5 Department of Obstetrics and Gynaecology, Carol Davila University of Medicine and Pharmacy, Bucharest, ROU

**Keywords:** mental health services, depression, anxiety, emotional distress, congenital heart disease

## Abstract

Congenital heart disease (CHD) is a common and significant congenital disorder. Despite advancements in neonatal care, Congenital heart disease remains a leading cause of death among infants with congenital malformations. Congenital heart disease is responsible for a significant portion of stillbirths and includes several forms with various anatomical variations. A CHD diagnosis leads to significant emotional distress for parents, affecting family dynamics and quality of life. Parents often experience psychological impacts, such as anxiety, depression, and guilt, particularly when diagnoses occur after birth. This literature review explores the psychosocial impact of CHD on families and examines how the timing of diagnosis influences outcomes. Parents face numerous challenges, including managing complex medical care and addressing financial and emotional burdens. Effective communication between healthcare providers and families is crucial, as is providing continuous emotional support and counseling. Early psychological interventions can alleviate depression and anxiety, and pediatric psychologists play a significant role in reducing the negative long-term effects on neurodevelopment. Further research is needed to develop strategies to improve mental health services and enhance the quality of life for families affected by CHD.

## Introduction and background

Congenital heart disease (CHD) is one of the more common and significant congenital disorders, affecting over one million newborns worldwide each year [1]. In Europe, the incidence of CHD is approximately 8.2 per 1,000 newborns, while in Africa, it is reported as only 1.9 per 1,000 live births, and in Asia, 9.3 per 1,000 live births [2]. These numbers may be underestimated, especially in low- and middle-income countries, where access to medical services and diagnostic investigations is limited and many miscarriages are caused by CHD [3]. The care of children with heart disease has increasingly become a neonatal specialty. Fetal diagnosis of CHD is now routine, and cardiovascular surgery is performed soon after birth and even in utero in selected cases [4].

Despite significant medical progress in diagnosing and treating these patients, the incidence of CHD has not decreased and is still considered the leading cause of death among infants born with congenital malformations [5]. More scientific research is necessary to advance the prevention of CHD, better predict its severity, and alleviate its consequences for children and adults [6].

Congenital heart disease is defined as a structural malformation of the heart and/or major vessels of the fetus that occurs during intrauterine development and is included in the category of the most debilitating chronic disorders in infants [7]. Congenital heart disease is responsible for 20% of stillbirths [8]. According to the American Heart Association, there are at least 18 forms of CHD with additional anatomic variations [9]. Table 1 presents the most common classification of CHD based on the presence of cyanosis.

**Table 1:** Classification of CHD according to the presence of cyanosis CHD: congenital heart disease

Acyanotic heart disease	Cyanotic heart disease
Atrial septal defect	Tetralogy of Fallot
Ventricular septal defect	Tricuspid atresia
Patent ductus arteriosus	Pulmonary stenosis/atresia
Aortic stenosis	Transposition of great arteries
Atrioventricular septal defect	Total anomalous pulmonary venous return
Coarctation of the aorta	Persistent truncus arteriosus
	Hypoplastic left heart syndrome
	Ebstein anomaly

Due to significant advances in surgical care for children with CHD, recent data have reported a significant improvement in survival rates, with rates reaching 80% to 95% in high-income countries. Therefore, CHD may be considered a chronic disorder rather than a life-threatening one [10]. Table 2 summarizes the classification of CHD according to short- and long-term prognosis and treatment [11].

**Table 2:** Classification of CHD according to short and long-term prognosis and treatment CHD: congenital heart disease; TAPVC: total anomalous pulmonary venous return

Category of CHD	Prognosis	Treatment	CHD
Critical	Incompatible with survival without early intervention	Early intervention in the first month of life	Transposition of great arteries; obstructed TAPVC Duct-dependent pulmonary or systemic circulation [11]
Major	Optimal long-term outcome	Intervention is required in early infancy	Tetralogy of Fallot; double outlet right ventricle; large ventricular septal defect /patent ductus arteriosus; complete atrioventricular canal; truncus arteriosus; severe outflow tract obstruction [11]
Asymptomatic at birth	Rarely diagnosed in early childhood, long-term sequelae prevented by specific intervention	Rarely diagnosed in early childhood	Moderate/large atrial septal defect; some forms of coarctation; some patients with Ebstein’s anomaly; relatively less severe forms of aortic and pulmonary valve stenosis [11]
Minor	Long-term, symptom-free survival	Any specific intervention in most cases	Small left-to-right shunts [11]

Early studies on the impact of fetal diagnosis of CHD on postnatal outcomes were not promising. However, more recent publications suggest there is an improvement in postnatal morbidity and mortality if the diagnosis is made earlier [12,13]. One study examining the impact of prenatal diagnosis in hypoplastic left heart syndrome showed that all patients diagnosed prenatally who underwent surgery survived, compared with only 65% of those diagnosed postnatally [14]. Another study on prenatal diagnosis in cases of hypoplastic left heart syndrome did not show a difference in overall mortality but revealed less neurologic impairment compared with patients diagnosed postnatally [15].

Giving birth to a child with CHD causes significant emotional distress, including shock, denial, fear, anxiety, and depression, which alters the parents’ perception of the childbirth experience. Current publications emphasize the need to understand the psychosocial impact on both children with CHD and their parents. Additionally, siblings in a family with a child suffering from CHD are also affected and may experience anxiety and depression [16]. The quality of life of these families is greatly diminished, primarily due to the infant’s suffering and the potentially fatal diagnosis. Not only is the psychosocial impact severe, but all aspects of life are impaired, and the financial burden exacerbates the level of stress and emotional pressure [17]. This research aims to explore the psychological impact of having an infant with CHD and the differences related to the timing of the diagnosis. We emphasize the difficult situations and the negative psychosocial impact on parents who must face the diagnosis of severe CHD, either prenatally or after birth. Medical professionals can also be affected by dealing with the emotional challenges of patients and their families; unfavorable courses of the disease can be distressing and frustrating. Future studies should focus on implementing specific interventions to support couples during prolonged hospitalization and after discharge.

## Review

For this research, we used the Google Scholar and PubMed databases to extract data from original articles, research articles, reviews, and protocols. We used terms such as “congenital heart disease,” “pregnancy with congenital malformation,” “negative psychosocial impact on parents,” and “pediatric psychologist,” including only articles published in English since 2017, as listed in the references section. We reviewed original articles that studied the experiences of families with infants diagnosed with CHD and the negative impact of the illness on their quality of life. This allowed us to evaluate the psychosocial impact on parents of infants with CHD and determine whether the timing of the diagnosis, either during pregnancy or after birth, influences significant differences in outcomes.

Facing the CHD diagnosis

When expectant parents are informed by the obstetrician during pregnancy about a CHD diagnosis in the fetus, they experience multiple psychological changes, including shock, denial, pain, guilt, and depression [18]. The tremendous technological progress in obstetrics, maternal-fetal medicine, and fetal echocardiography now leads to early and highly accurate diagnoses of cardiac malformations during pregnancy in most cases [19]. Consequently, the medical team can provide accurate information about the fetus, diagnosis, delivery, prognosis, and postnatal surgical therapies [20]. However, despite these advances, the cause of CHD remains unclear in most pregnancies, often leaving families overwhelmed by frustration and guilt, along with the feeling that they will be unable to cope with the situation [21]. Occasionally, a prenatal diagnosis may underestimate the problem, or associated issues (such as prematurity, infection, or other congenital anomalies) may delay or alter the initial treatment plan. This can generate impatience, mistrust, and confusion for parents, making it essential for medical professionals to be responsive and supportive. Overall, a CHD diagnosis means grief and stress at that time, requiring the family to adjust. Awareness of the psychological impact and providing health counseling by professionals can improve levels of depression, stress, and anxiety [22].

Epidemiological studies have suggested that CHD may have genetic control, and the risk of recurrence in siblings or offspring of parents with CHD is possible in 3% to 5% of cases. This can be frightening for young couples facing a first pregnancy with fetal CHD [23]. The fetal or neonatal expression of these disorders or their particular phenotype shows great variability [24].

If a severe CHD is diagnosed during the first trimester of pregnancy, parents have the option to decide on the termination of the pregnancy. Religious beliefs or moral judgments may oppose this decision, and the burden of responsibility, combined with fear, can destabilize the couple’s life. Mothers usually find it more difficult to decide on an abortion compared to fathers, and differences of opinion in this context can lead to unwanted tensions. Medical teams (obstetricians, neonatologists, cardiologists, and cardiac surgeons) should provide all necessary medical data to help parents understand the prognosis of the disease, enabling them to make an informed decision. It is important to support the family in their choices and, above all, avoid being judgmental. Beyond statistics and numbers, parents should be told that each child is unique, and the clinical course of the same disease may differ among patients; a certain prognosis is better not given.

Several studies have emphasized the importance of the timing of diagnosis, prenatal versus postnatal [21]. The earlier future parents become aware of the congenital disorder, the sooner they can start the process of accepting the situation and preparing for the implications of the disease after delivery [25]. With access to a large amount of information, they can learn about their child’s vulnerabilities and needs, identify symptoms that may indicate a worsening condition, and direct their thoughts and energy toward a useful purpose. Parents who are informed about the diagnosis after birth are overwhelmed by negative emotions and are unable to make rapid, rational decisions. Sometimes, they have only a short period until surgery, requiring them to quickly adapt to the medical condition and consequences amid great distress. A study by Bevilacqua et al. showed that mothers informed about the diagnosis during pregnancy suffered primarily from depression, while mothers who received the CHD diagnosis after delivery experienced a great deal of stress [26]. Unfortunately, high levels of anxiety and distress during pregnancy are correlated with preterm birth and intrauterine growth restriction [27]. Postnatal stress interferes with lactation and bonding with the newborns and may exacerbate postpartum hormonal depression. Thus, intervention from psychologists may have a beneficial outcome, although there is limited research on this specific topic.

Parents of CHD newborns admitted to the neonatal intensive care unit (NICU)

Once the decision to continue the pregnancy is made, the obstetrician must advise the mother to deliver at a tertiary center that can provide all the necessary care facilities for children with CHD. This approach minimizes the need for newborn transfer to another hospital after birth for further interventions, as transport can be aggravating. It also helps prevent family separation from the child. In these life-threatening situations, it is beneficial for the parents to be familiar with the hospital and medical team (prenatal meetings are highly important). Providing a schedule of future interventions and treatment can alleviate the fear of the unknown and prepare them for unexpected and unfortunate events.

After delivery, parents must navigate the transition to parenthood while managing the diagnosis of CHD and related psychological, medical, economic, and social issues [28]. The NICU can be a frightening and traumatizing experience for mothers, with many reporting fear of their newborn dying during admission and experiencing significant trauma symptoms by discharge [29]. Existing evidence demonstrates that mothers of cyanotic CHD newborns suffer from significantly higher levels of depression than mothers of acyanotic infants [30]. Restrictions imposed in the NICU regarding visitation schedules and physical contact with the baby generate high levels of stress, anxiety, and a lack of control for parents [29]. In addition, during prolonged hospitalization, mothers’ physical health can be affected by poor nutrition, exhaustion, and disrupted sleep.

When facing the reality of not having a healthy newborn, all family members experience high levels of distress, manifested in various forms such as depression, fear, anxiety, hopelessness, and somatization. A study by Dale et al. revealed that mothers of CHD infants are at risk of developing mental illness [31]. The negative emotional impact can lead to acute stress disorder and post-traumatic stress disorder (PTSD) [32]. Recent research shows that parents with PTSD may have children who develop eating and sleeping disorders and have more frequent visits to the emergency department [32].

Some mothers report difficulties bonding with their children and find the feeding process a source of frustration and distress [33]. Stress, lack of physical contact, and insufficient water intake can decrease milk secretion. Poor attachment with the sick newborn, along with prolonged hospitalization, can compromise parenting abilities and self-confidence, risking short- and long-term psychological issues. The levels of stress correlate with the severity of CHD [33].

If the complexity of CHD necessitates surgical interventions at a different hospital, the newborn may be referred to a specialized center, sometimes in another country. Administrative procedures, travel, language barriers, unfamiliar medical staff, a new environment, and being away from the rest of the family add stress that amplifies fear and insecurity. Non-governmental organizations, patient support groups, and credible medical staff with good communication skills can significantly help in this situation.

In striving for normalcy, families integrate CHD into their lives in their own way, depending on prior experience, personal characteristics, family beliefs and religion, and social and financial support availability.

Infants with CHD

Admittance to the NICU subjects infants to enormous stress levels due to the environment, which can have a long-term impact on their neurodevelopment [34]. Both the physical and medical environments in the NICU are unfavorable for newborn development, especially preterm neonates. Lights, sounds, noises, and odors, along with repeated medical procedures and continuous monitoring of vital signs, exacerbate neuropsychic and physical impairment [35]. Furthermore, additional stressors include medication, mechanical ventilation, nasogastric tubes, central line placement, analgesia, and blood sample collection [36]. Adverse effects of some drugs, hospital-acquired infections, pain from medical procedures, anemia due to frequent phlebotomies, and sensory deficiencies can represent complications of long-term hospital admission. Beyond the effects of CHD, sedation, analgesia, immobility, and lack of maternal presence may also interfere with neurological outcomes. Prolonged hospitalization in the NICU decreases parent-infant attachment due to limited bonding time and interaction with caregivers [37].

Congenital heart disease, combined with stressful factors encountered from birth, leads to neurobehavioral delays and abnormalities in over 50% of newborns [38]. Current data confirm that arterial oxygen saturation below 85% predicts neurodevelopmental impairment. Moreover, complex surgical procedures may contribute to brain injury [39].

Numerous risk factors can affect the neuropsychic development of newborns with CHD, ranging from maternal stress during pregnancy to cardiorespiratory resuscitation at birth and associated comorbidities during prolonged hospitalization [40]. Regarding intelligence and cognitive function during the school-age period, studies show noticeable differences between children who experience hypothermic cardiopulmonary bypass and those with less severe CHD [41]. New studies motivated by the desire to better understand the psychosocial needs of children with CHD focus on highlighting the importance of therapy as part of the treatment, alongside surgical interventions [42].

Living with a chronic illness with an unpredictable prognosis can negatively impact the psychological development of children born with CHD, especially during adolescence [43]. It is difficult for them to come to terms with the illness, and it is overwhelming and exhausting to deal with repeated medical procedures. Furthermore, they often feel helpless and disappointed when exacerbations occur. Research conducted by Karsdorp et al. indicates that adolescents are at risk of developing internalizing issues, such as anxiety or depression, as well as externalizing problems, such as aggressive or hyperactive behavior [44].

Going home with a newborn with CHD: fears and concerns

Congenital heart disease often means a lifetime of stress, recurrent hospital admissions, and painful surgeries and procedures, often more than one, along with lifelong treatment. This affects the children and the entire family's dynamics, organization, and functioning [45]. Upon discharge, families encounter additional stressors, compounded by the constant uncertainty of the infant’s survival [46]. Most infants who undergo CHD surgery experience feeding difficulties and weight gain problems. After open-heart surgery, dysphagia occurs in up to 20% of cases [47]. More than 50% of these infants may be discharged with feeding tubes or special devices, requiring additional practical training for caregivers [48]. Procuring medical devices can be stressful and resource-demanding, and providing home care can be challenging in some situations.

An important issue is the responsibility for supervising home care for children with CHD before or after surgery. General practitioners and pediatricians should communicate with cardiologists for ongoing treatments and recommend all necessary prophylactic interventions.

Discharging a newborn with CHD scheduled for later surgery is a difficult decision, as the benefits of the home environment must be balanced with safety. While parents are happy to have their children at home, they will experience high levels of uncertainty and anxiety. It is important to provide them with clear instructions and a personalized follow-up plan for the baby, assuring them of constant support to increase self-confidence and reduce insecurity.

For progressive, life-limiting CHD with a poor prognosis, referral to palliative care centers can alleviate parents’ grief by allowing them to be close to their infants and provide comfort throughout their final days. The medical team should gently discuss this option with parents as an alternative for providing treatment, symptom management, and pain-free procedures. During repeated examinations, medical staff involved in the infant’s care should also be attentive to parental stress, inquiring about family function and their specific needs. Unfortunately, the marital status may be affected as all attention focuses on the sick child’s needs, reducing satisfaction and increasing the couple’s vulnerability.

Undoubtedly, the negative psychosocial impact on caregivers of children with CHD includes feelings of anxiety, panic, stress, fear, depression, uncertainty, and guilt that can persist for a long period [49]. Some studies found these feelings can persist up to 50 months after surgery [50]. Another finding is the difference in emotional impact between mothers and fathers; studies highlight that mothers bear more of the burden and suffer higher levels of distress [51]. Further research on paternal data is needed as specific information is sparse. Therapy focused on fathers’ involvement in infant care may improve engagement and efficacy.

Parents often neglect their own mental and physical health, prioritizing their infant’s well-being. Several studies have examined how parents cope with their infants’ medical conditions. To adapt and reduce the distress of CHD in daily life, parents adopt various strategies. For many, extended family, especially grandparents, offer great emotional support. Connecting with families who share similar experiences and enrolling in peer support groups can help them cope with their emotions.

In some cases, faith and religion provide comfort in dealing with the situation. Avoidance and denial are coping styles seen in parents who struggle to accept the CHD diagnosis shortly after receiving the news [52]. Isolation, avoiding communication, constant self-sacrifice, and imposing several restrictions on daily activities tremendously negatively affect the quality of life.

Diagnosis communication

Communicating a diagnosis and discussing prognosis and life-threatening issues with the family can be difficult and uncomfortable for many healthcare professionals. Reasons for this include lack of training, inability to attend to parents’ emotional needs, and awareness of their feelings of anxiety and sadness. The balance between honesty and hope must rely on accurate information, with healthcare professionals consulting the literature to obtain up-to-date knowledge about the diagnosis [53]. Information about illness progression, life expectancy, associated risks, future symptoms, and end-of-life issues can be difficult for families to comprehend all at once, necessitating further discussions. It is essential to clarify to parents that prognosis predictions are uncertain and unreliable. Recognizing the impact of the information on the family and communicating with empathy and understanding will help ensure a relationship based on trust between families and healthcare professionals.

Each parent confronted with a severe CHD diagnosis finds their coping mechanisms, going through a complex psychological process that requires support and guidance from the extended family, society, and especially from medical staff and psychologists. Understanding the experiences of families with CHD will facilitate the implementation of future interventions that aim to decrease the negative impact and psychosocial consequences experienced by children with CHD and their caregivers.

Meanwhile, healthcare professionals must focus on assessing parents’ emotional status and spiritual needs, providing them with medical information, and providing empathetic and individualized support. They must encourage families to accept counseling to develop their own coping mechanisms. It is important to start psychological intervention for parents while the newborn is in the NICU, as this can reduce depression and anxiety.

Furthermore, psychologists play a significant role during childhood and adolescence, helping to reduce negative long-term effects on the child’s neurodevelopment [54]. Physical limitations often lead to social exclusion, discrimination, or bullying, making CHD a limiting factor for several experiences. These children face the difficulty of coping with the illness and lack self-confidence and hope for improvement [55]. With the help of school psychologists and teachers, adequate cognitive behavioral therapy can improve the quality of life for these children and their families. The beneficial role of psychologists is also evident during the transition from adolescence to adulthood when CHD is still perceived as a stressful factor [40]. Media can also play an important role in raising awareness about CHD and encouraging screening and early detection by reaching out to prenatal healthcare institutions.

## Conclusions

This review highlights the mental health burden on parents of infants with CHD and the challenges they face while struggling with a life-threatening condition. Most parents of infants with CHD experience a negative psychosocial impact, regardless of whether the diagnosis is made during pregnancy or after birth. The difference is that parents who receive the diagnosis during pregnancy may experience anticipatory grief and anxiety, while parents who receive the diagnosis after birth may feel shock and disbelief. Further work is needed to develop strategies that provide adequate mental health services to improve their quality of life.
